# Quercetin induces autophagy-associated death in HL-60 cells through CaMKKβ/AMPK/mTOR signal pathway: Quercetin induces autophagic cell death in AML

**DOI:** 10.3724/abbs.2022117

**Published:** 2022-09-19

**Authors:** Jie Xiao, Ben Zhang, Songmei Yin, Shuangfeng Xie, Kezhi Huang, Jieyu Wang, Wenjuan Yang, Hongyun Liu, Guoyang Zhang, Xiaoyan Liu, Yiqing Li, Danian Nie

**Affiliations:** 1 Department of Hematology Sun Yat-Sen Memorial Hospital Sun Yat-Sen University Guangzhou 510120 China; 2 Guangdong Provincial Key Laboratory of Malignant Tumor Epigenetics and Gene Regulation Sun Yat-Sen Memorial Hospital Sun Yat-Sen University Guangzhou 510120 China; 3 Department of Cardiothoracic Surgery General Hospital of Southern Theatre Command the People’s Liberation Army Guangzhou 510010 China

**Keywords:** acute myeloid leukemia, quercetin, autophagy, AMP-activated protein kinase, mammalian rapamycin target protein

## Abstract

Acute myeloid leukemia (AML) is one of the most common malignancies of the hematopoietic progenitor cell in adults. Quercetin has gained recognition over the years because of its anti-cancer effect with minimal toxicity. Herein, we aim to investigate the anti-leukemia mechanism of quercetin and to decipher the signaling pathway of quercetin in HL-60 leukemic cells. We observed that quercetin induces apoptosis and autophagic cell death, in which both pathways play an important role in suppressing the viability of leukemia cells. Phosphorylated AMPK (p-AMPK) protein expressions are lower in primary AML cells, HL-60 cells, KG-1 and THP-1 cells than in peripheral blood monocular cells. After quercetin treatment, the expression of p-AMPK is increased while the expression of p-mTOR is decreased in a dose-dependent manner. Mechanistically, compound C, an AMPK phosphorylation inhibitor, upregulates the phosphorylation of mTOR and inhibits autophagy and apoptosis in quercetin-induced HL-60 cells, while silencing of CaMKKβ inhibits the quercetin-induced phosphorylation of AMPK, resulting in increased mTOR phosphorylation. Furthermore, silencing of CaMKKβ inhibits the autophagy in HL-60 cells. Taken together, our data delineate that quercetin plays its anti-leukemia role by inhibiting cell viability and inducing apoptosis and autophagy in leukemia cells. Quercetin inhibits the phosphorylation of mTOR by regulating the activity of AMPK, thus playing a role in the regulation of autophagy and apoptosis. CaMKKβ is a potential upstream molecule for AMPK/mTOR signaling pathway, through which quercetin induces autophagy in HL-60 cells.

## Introduction

Quercetin belongs to flavonoid family and is almost non-toxic. As a type of OTC, quercetin is used for the treatment of prostate cancer [1]. The structure of quercetin is stable and quercetin is easy to be separated and purified. Due to its antioxidant and anti-cancer effect, quercetin has gained recognition over the past years [ 2, 3] . It is a type of “molecular targeted drug” that can interfere with the signal transduction pathway of tumor cells [ 4, 5] . It was found that flavonoids, including quercetin, can inhibit the proliferation of many hematologic malignant tumor cells, including leukemia cells [ 6, 7] . Our previous study also revealed that quercetin can inhibit the proliferation of HL-60 cells and induce apoptosis [8]. The in-depth mechanism, however, remains ambiguous.

Autophagy is a kind of programmed cell death, an emerging form of cell death found in cancer [9]. Induction of autophagy may be a new method for acute leukemia therapy [ 10, 11] . However, some studies suggest that autophagy plays a protective role in the development of leukemia [7]. Whether quercetin can exert its antitumor activity through inducing autophagy-associated death of acute myeloid leukemia (AML) cells has not been reported. Autophagy is closely related to cell energy metabolism [12]. AMP-activated protein kinase (AMPK) is a central molecule that regulates cell energy metabolism, and it is also a regulatory molecule in the signal pathway of autophagy [ 13, 14] . Tumor is often accompanied by energy metabolism disorder and AMPK activation inhibition. AMPK signaling pathway is closely related to the occurrence of autophagy [ 15, 16] . Mammalian rapamycin target protein (mTOR) is an important downstream signal molecule of AMPK and participates in the progress of tumors. Over-activation of mTOR can accelerate the cell cycle of tumor cells and promote cell migration and apoptosis-resistance [ 17, 18] . As an allosteric molecule, the complete activation of AMPK also requires the phosphorylation of the threonine (threonine 172) in its catalytic center by the upstream kinases. The upstream kinases of AMPK include LKB1, CaMKKβ, and TAK1 [ 19– 21] . In our previous study, we observed that quercetin could activate AMPK and inhibit the proliferation of HL-60 cells [8]. Further study is needed to elucidate the mechanism of quercetin’s anti-leukemic function.

In this present study, we attempted to seek novel drug targets in the treatment of AML and provide the theoretical foundation for the development of anti-cancer drugs with low toxicity. We found that quercetin could induce apoptosis and autophagy in acute leukemia cells. Inducing the phosphorylation of AMPK or inhibiting the mTOR activity in leukemia cells may be a potential method for acute leukemia treatment. Quercetin may activate AMPK and inhibit the phosphorylation of mTOR, thus inducing apoptosis and autophagy in HL-60 cells. CaMKKβ siRNA could effectively inhibit the protein expression of CaMKKβ. When CaMKKβ gene was silenced in HL-60 cells, the quercetin-activated AMPK phosphorylation was decreased, and its ability to inactivate mTOR function was reduced. CaMKKβ siRNA had a negative effect on the ratio of LC3 II/I which was upregulated by quercetin. We propose that quercetin induces autophagy-associated death in HL-60 Cells through CaMKKβ/AMPK/mTOR signal pathway.

## Materials and Methods

### Cells and cell culture

Human AML cell lines HL-60, THP-1, and KG-1 were purchased form Tianjin Institute of Hematology of the Chinese Academy of Medical Sciences (Tianjin, China). Cells were seeded in RPMI 1640 containing 10% fetal bovine serum and 3% glutamine, cultured in an incubator at 37°C with 5% CO _2_, and passaged with medium changed every 2 or 3 days. The cells in the logarithmic growth phase were used for the subsequent experiments.

Normal human PBMCs were collected from twelve healthy subjects who underwent routine physical examinations in Sun Yat-sen Memorial Hospital of Sun Yat-sen University. AML primary cells were obtained from thirty-eight AML patients in our hospital without M3 French-American-British (FAB) Classification **.** The research methods were approved by the Ethics Committee of Sun Yat-sen Memorial Hospital of Sun Yat-sen University and an informed consent statement was signed by each participant. Blood or bone marrow was treated with ethylenediamine tetracetic acid for anti-coagulation, diluted with 2-fold volume of phosphate-buffered saline (PBS, pH 7.2), and mixed well. The cell suspension was added with caution to the lymphocyte separation liquid equal in volume to the blood, and centrifuged horizontally at 500 *g* at room temperature for 20 min. The PBMCs at the junction of the plasma layer and the lymphocyte separation liquid were collected, followed by addition of equal amount of PBS, mixed well, and centrifuged at 500 *g* for 10 min. After the supernatant was discarded, the cells were washed twice to remove the residual lymphocyte separation liquid.

### Materials

Autophagy detection kit, rabbit anti-human LC3B IgG polyclonal antibody, mouse anti-human GAPDH IgG monoclonal antibody, rabbit polyclonal to LC3B, mouse monoclonal anti-AMPK alpha 1+AMPK alpha 2 antibody, rabbit monoclonal anti-AMPK alpha 1 (phospho T183)+AMPK alpha 2 (phospho T172) antibody, rabbit monoclonal anti-CaMKK antibody, rabbit polyclonal anti-LKB1 antibody, rabbit polyclonal to anti-LKB1 (phospho S428) antibody, rabbit monoclonal anti-mTOR antibody, rabbit monoclonal anti-mTOR (phospho S2448), rabbit monoclonal anti-TAK1 antibody, and mouse monoclonal anti-GAPDH antibody were purchased from Abcam (Cambridge, UK). Quercetin was from Sigma (St Louis, USA) and compound C was from Merck (Darmstadt, Germany).

### Cell proliferation and cell apoptosis analysis

The cells were incubated with different concentrations of quercetin (0, 25, 50, 75, and 100 μM) for 24, 48 and 72 h. Cell viability was detected by CCK-8 assay as described previously [8]. Colony formation assay which is used to evaluate the proliferative potential of cells was performed as described previously [22].

For apoptosis detection, the cells were washed twice with PBS and diluted to a final concentration of 1×10 ^6^ cells/mL, and then incubated with Annexin V-fluorescein isothiocyanate (FITC) and propidium iodide (PI) for 30 min in the dark. The cell apoptosis was determined by Flow Cytometry on a BD flow cytometer (Becton-Dickinson, Temse, Belgium). Early stage of cell apoptosis was defined as Annexin V-FITC ^+^PI ^–^ and late stage as Annexin V-FITC ^+^PI ^+^.

### Electron microscopy

The cells were harvested and fixed with 2.5% glutaraldehyde and 2% paraformaldehyde in sodium cacodylate buffer (pH 7.2) at 4°C. The specimens were then fixed in 0.5% osmium tetroxide (OsO4) for 30 min at 4°C, dehydrated using an ethanol gradient (50%, 60%, 70%, 80%, 90%, and 100% for 20 min each), and transferred to EM812 medium (EMS). After impregnation with pure resin, the specimens were embedded in the same resin mixture. The samples were sectioned (60 nm) with an ultramicrotome (UC7; Leica Microsystems, Wetzlar, Germany), collected on nickel grids, and then stained with uranyl acetate and lead citrate in a Leica EM Stainer (Leica Microsystems). The products were examined by electron microscopy at the Faculty of Medical Sciences of Sun Yat-sen University (Guangzhou, China) and the autophagosomes were imaged and analyzed.

### Immunofluorescence (IF) microscopy

Cellular autophagy was detected using an Autophagy Detection Kit (ab139484; Abcam). After treatment, the cells were washed twice with 1×assay buffer. The supernatant was carefully removed and 100 μL of microscopy dual detection reagent solution was dispensed to cover the cell pellet. The samples were protected from light and incubated for 30 min at 37°C. The cells were washed with 1×assay buffer and then resuspended in 100 μL 1×assay buffer. A drop of the cell suspension was applied onto a glass microscope slide and overlaid with a cover slip. The stained cells were analyzed under a wide-field fluorescence microscope (Olympus, Tokyo, Japan). A standard FITC filter set was used to image the autophagic signal, and a DAPI filter set was used to image the nucleus.

### Flow cytometry

Cell autophagy was also detected by Flow Cytometry. After being washed with 1×assay buffer, the cells were resuspended in 250 μL of indicator free cell culture medium containing 5% FBS and 250 μL of the diluted Green stain solution, and then incubated for 30 min at room temperature or 37°C in the dark. After collection and fixation, the cells were incubated for 20 min with 10% formalin and washed 3 times with 1×assay buffer. Flow Cytometry (Becton Deckinson, Franklin Lakes, USA) was used to quantify cell autophagy with FL1 channel.

### siRNAs and cell transfection

The design and synthesis of LKB-1, CaMKKβ, and TAK1 siRNA were completed by GenePharma Co., Ltd (Shanghai, China). The nucleotide sequences of sense and antisense LKB-1, CaMKKβ, and TAK1 siRNA were listed in Table 1. FAM-labeled negative control siRNA was provided by Lonza Co. (Catalog No. VCA-1003; Basel, Switzerland), which did not match any gene of known function in GenBank.

**Table:** **Table 1** The nucleotide sequences of siRNAs used in this study

Name	Sense (5′→ 3′)	Antisense (5′→ 3′)
LKB-1 siRNA	CCUGCUGAAAGGGAUGCUUTT	AAGCAUCCCUUUCAGCAGGTT
CaMKK-β siRNA	GCAUCGAGUACUUACACUATT	UAGUGUAAGUACUCGAUGCTT
TAK-1 siRNA	GUCCCAGUGUCAGAAUGAUTT	AUCAUUCUGACACUGGGACTT

One nucleofection sample contained 2×10 ^6^ cells, 10 mL of 20 mM siRNA and 100 mL Amaxa Cell Line Nucleofector Solution V (Catalog No. VCA-1003; Lonza Co). The supplemented Nucleofector Solution V was pre-warmed to room temperature. The 6-well plates filled with culture medium containing supplements and serum were pre-incubated in a humidified 5% CO _2_ incubator at 37°C. The cells were resuspended in Cell Line Nucleofector Solution V to a final concentration of 2×10 ^6^ cells/ 100 mL nucleofection. The transfection program was started by mixing the nucleofection sample with 10 ml siRNA and then transferred to an Amaxa certified cuvette. After the program was completed, the sample was transferred to the prepared 6-well plates. Cells were incubated in a humidified incubator at 37°C with 5% CO _2_.

### Reverse transcription-polymerase chain reaction

Total RNA was isolated from the cells in each group using Trizol regent (Invitrogen, Carlsbad, USA) according to the manufacturer’s instruction. Total cellular RNA was used for reverse transcription (RT) of cDNA by a standardized technique (Takara, Japan). Obtained cDNA was amplified using specific primers listed in Table 2. After pre-denaturation at 95°C for 30 s, polymerase chain reaction (PCR) was carried out for 30 cycles: 10 s denaturation at 95°C, followed by annealing for 20 s at 60°C and finally extended for 30 s at 72°C. Realtime PCR was performed on the Bio-Rad CFX96 Real Time PCR Instrument (Bio-Rad, Hercules, USA).

**Table:** **Table 2** The sequence of primers used in reverse transcription-polymerase chain reaction

Gene	Primer sequence (5′→ 3′)
*β-Actin*	Forward Reverse	TGGCACCCAGCACAATGAA CTAAGTCATAGTCCGCCTAGAAGCA
*LKB-1*	Forward Reverse	AGGGCCGTCAAGATCCTCAA CACACGCAGTACTCCATCACCA
*CaMKK beta*	Forward Reverse	GAAGACCTGGCCCGTTTCTACT TGCCCTTGAATTCATTGCTCAC
*TAK-1*	Forward Reverse	GCCTGATGACTCGTTGTTGGTCTA ATGGCTCATCTGCTCCTGGAA

### Western blot analysis

Total protein was extracted using RIPA lysis buffer (CWbiotech, Beijing, China) containing 1 mM phenyl methyl sulfonyl fluoride (PMSF; Beyotime, Shanghai, China). The protein concentration was measured using BCA Protein Assay kit (Beyotime). For western blot analysis, equal amounts of soluble protein (50 μg per well) were subjected to 12% SDS–polyacrylamide gel electrophoresis (SDS-PAGE) and transferred onto polyvinylidene difluoride (PVDF) membranes. Membranes were blocked with 5% skimmed milk at 37°C for 1 h and then incubated with the primary anti-LC3, TAK1, LKB1, CaMKK β, AMPK, p-AMPK, mTOR, and p-mTOR antibodies (Abcam, Cambridge, UK) on the rocking bed overnight at 4°C. After three times washe with TBST, the PVDF membranes were incubated with horseradish peroxidase-labeled goat anti-mouse IgG monoclonal antibody or goat anti-rabbit IgG monoclonal antibody in the blocking solution (1:10,000) at room temperature for 1 h. After being washed with TBST, membranes were detected using a DBA kit (Pulilai, Beijing, China). The images were analyzed using the gel analysis software (Quantity one; Bio-Rad, USA). All experiments were repeated three times.

### Statistical analysis

Data are expressed as the mean±standard deviation (SD). Comparison of the mean values of two independent specimens was conducted using Student’s *t*-test. Comparison of the mean values between the multiple groups was conducted using one-way analysis of variance or two-way analysis of variance. All data were treated using SPSS 20.0 statistical software (SPSS, Chicago, USA). *P*<0.05 was considered statistically significant.

## Results

### Quercetin inhibits cell viability and induces cell apoptosis in AML cells

**Figure 1:**
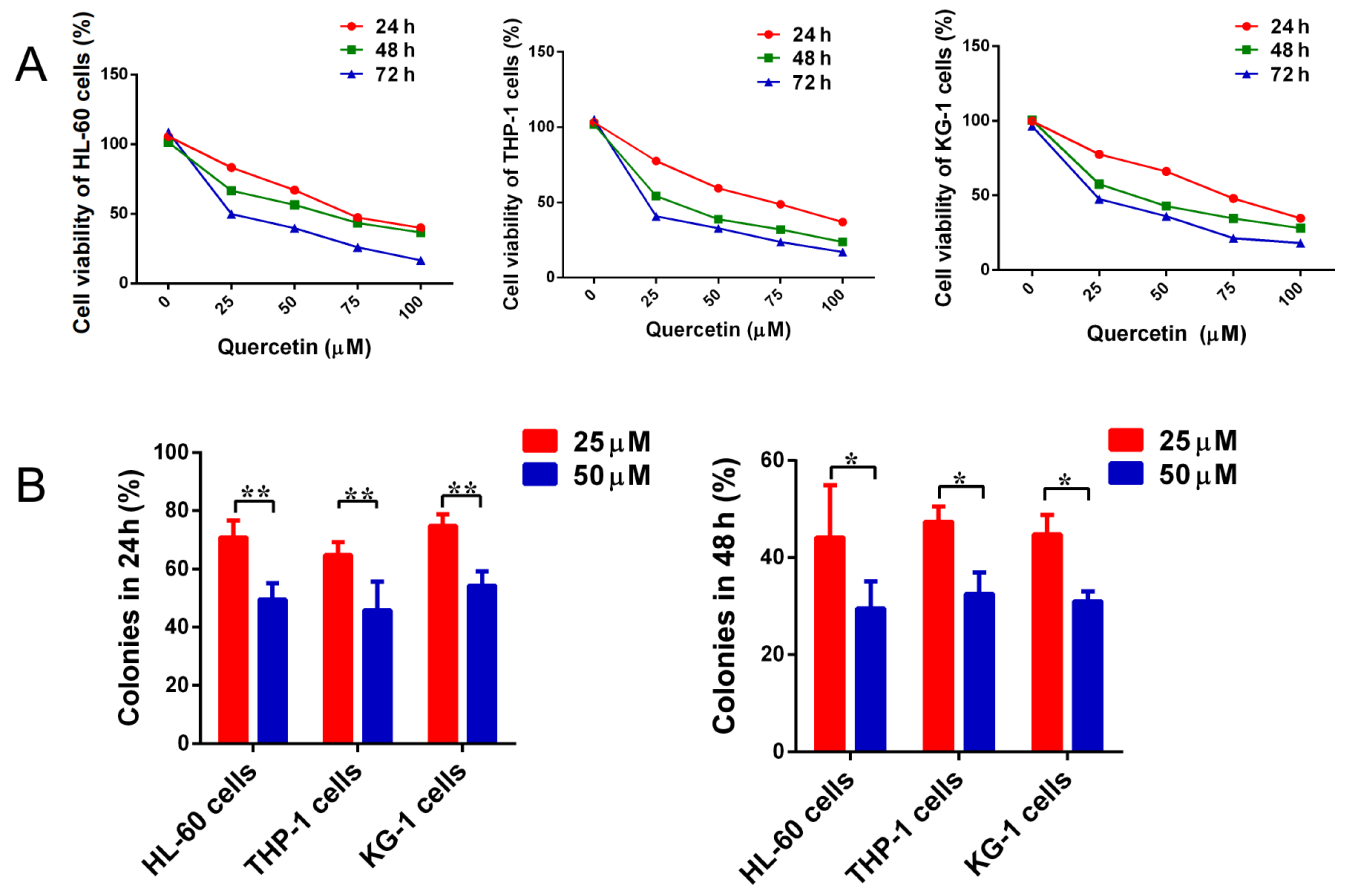
Figure 1 The effects of quercetin on the proliferation of HL-60, THP-1, and KG-1 cells (A ) The viabilities of HL-60, THP-1 and KG-1 cells were tested by CCK-8 assay. n=4 for each group. (B) The colony formation units of HL-60, THP-1 and KG-1 cells incubated with quercetin were calculated and showed. n=3 for each group. * P<0.05, ** P<0.01.

**Figure 2:**
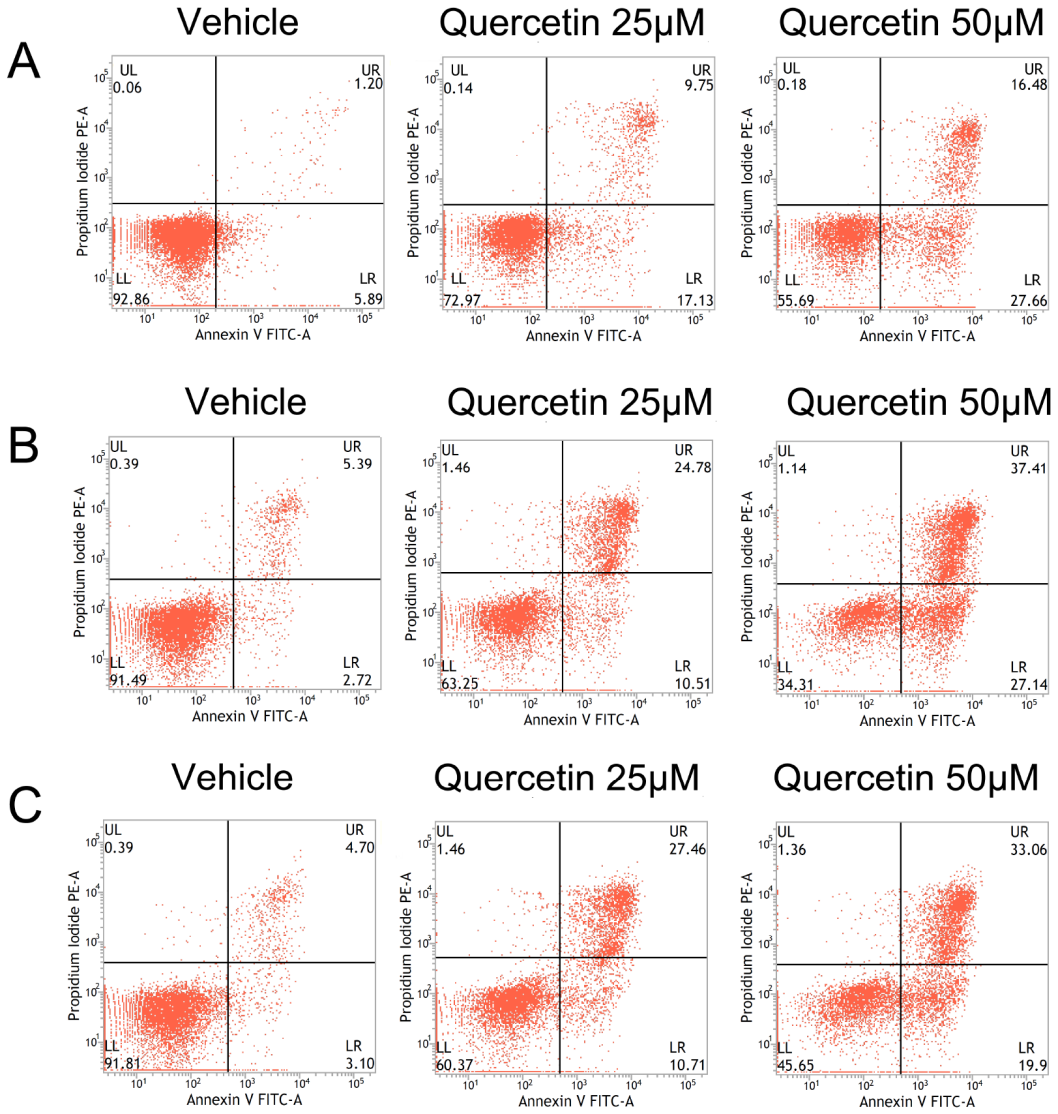
Figure 2 The apoptosis of HL-60 cells incubated with quercetin The apoptosis rates of HL-60, THP-1 and KG-1 cells incubated with or without quercetin were detected by flow cytometry. The early apoptosis was defined as PI (–) AnnexinV-FITC (+), late apoptosis was defined as PI (+) AnnexinV-FITC (+). (A) HL-60 cells. (B) THP-1 cells. (C) KG-1 cells.

HL-60, THP-1 and KG-1 cells were incubated with different concentrations of quercetin (0, 25, 50, 75, and 100 μM) for 24, 48 and 72 h. CCK-8 assay was used to detect the cell viability. The results showed that quercetin could effectively inhibit the viability of AML cells in a concentration-dependent manner ( Figure 1A). In addition, quercetin markedly suppressed colony formation of HL-60, THP-1 and KG-1 cells. Colony formation was decreased by the increase of quercetin concentration ( Figure 1B). The cell apoptosis was determined by flow cytometry. Quercetin induced apoptosis in HL-60, THP-1 and KG-1 cells. The apoptosis rate was increased with the increase in quercetin concentration ( Figure 2). These results showed that quercetin could inhibit proliferation and induce apoptosis in AML cells.

### Quercetin induces autophagy in AML cells

**Figure 3:**
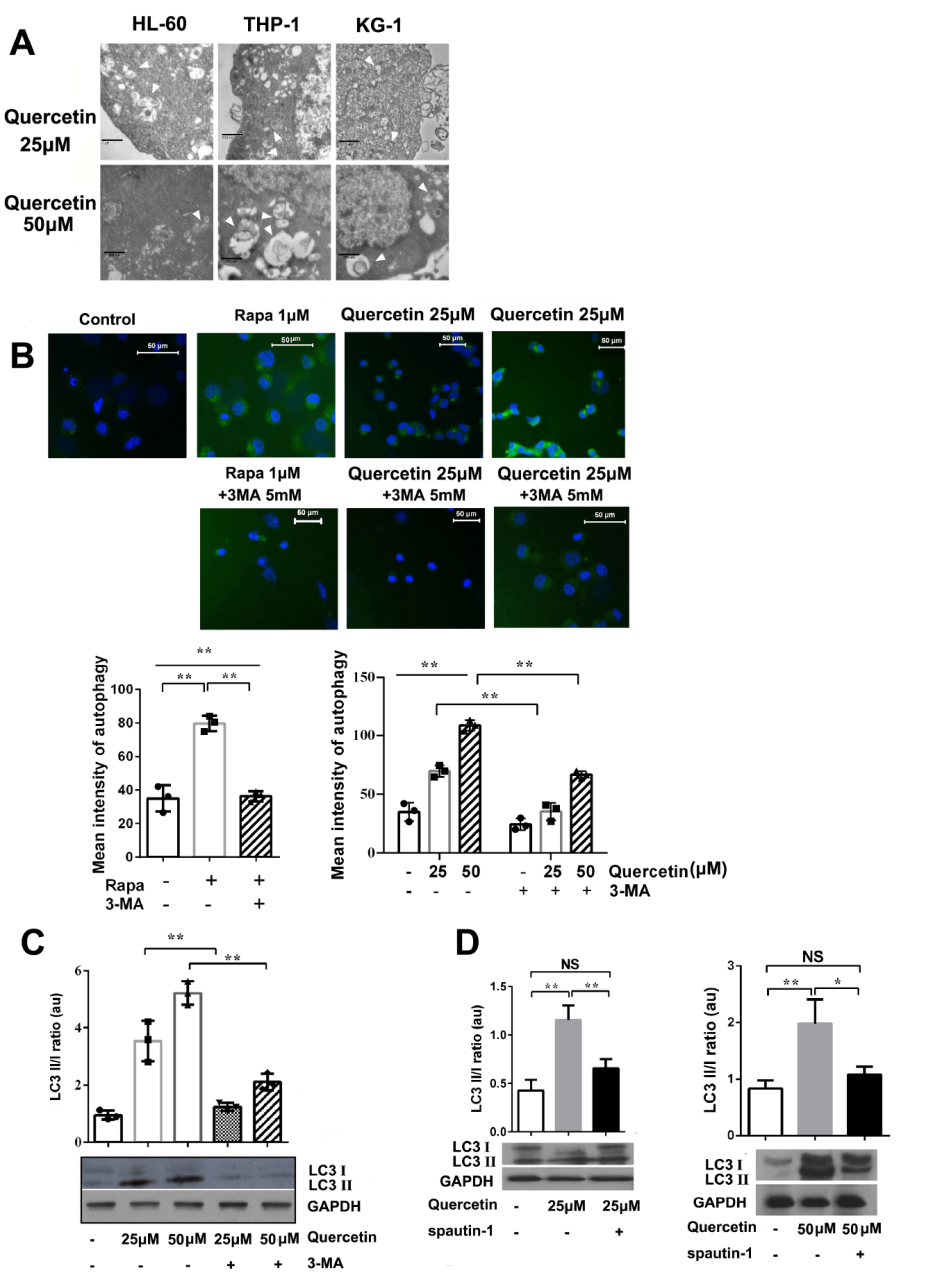
Figure 3 Quercetin induces autophagy in cells (A) HL-60, THP-1 and KG-1 cells were incubated with quercetin (25, 50 μM) for 48 h. The autophagosomes were observed by electron microscopy. Scale bars: 1 μm (upper right), 2 μm (upper left), 800 nm (upper middle, and lower). (B) The cells were incubated with quercetin (25 and 50 μM) , Rapamycin (1 μM), Rapamycin (1 μM)+3-MA (5 mM), quercetin (25, 50 μM)+3-MA (5 mM) for 48 h, respectively. The autophagy in HL-60 cells were detected by immunofluorescence microscopy and flow cytometry. Green detection reagent typically accumulated in spherical vacuoles in the perinuclear region of the cells and the foci distributed throughout the cytoplasm. Flow cytometry-based profiling of autophagy. Scale bar: 50 μm. n=3 for each group. ** P<0.01. (C) Western blot analysis was performed to analyze the expressions of LC3 II/I in HL-60 cells treated with quercetin only or in combination with 3-MA for 48 h. (D) Western blot analysis was performed to analyze the expressions of LC3 II/I in HL-60 cells treated with quercetin only or in combination with spautin-1 for 48 h. Data are presented as the mean±SD of 3 independent experiments. * P<0.05, ** P<0.01. NS, no significance.

HL-60, THP-1 and KG-1 cells were incubated with different concentrations of quercetin (25 and 50 μM) for 48 h. The cells were harvested and fixed with glutaraldehyde and examined by electron microscopy. The EM results showed that autophagosomes appeared in the cells after quercetin treatmnent ( Figure 3A).

Cell autophagy was also detected using Autophagy Detection Kit. Immunofluorescence microscopy and flow cytometry were applied to observe the autophagosomes and profile the autophagy in HL-60 cells. The results showed that cells in negative control group did not have green staining, while rapamycin (Rapa)-treated cells had intense punctuate structures. Flow Cytometry results showed that the average fluorescence intensity of the Rapa group was significantly higher than those of the control group and the Rapa+3-Methyladenine (3-MA) group. Quercetin could also induce auto- phagy in HL-60 cells. With the increase of quercetin concentration, the green fluorescence signal intensity was enhanced. The fluorescence microscopy results also showed that the staining became faint in the cells incubated with 3-MA+quercetin ( Figure 3B), indicating that 3-MA inhibited autophagy which was induced by quercetin or Rapa.

Western blot analysis results showed that the ratio of LC3 II/I in quercetin groups were higher than that in negative control group. Quercetin-induced cell autophagy could be inhibited by 3-MA ( Figure 3C). Spautin -1 (5 μM), another autophagy inhibitor, consistently reversed the ratio of LC3 II/I which was promoted by quercetin ( Figure 3D).

### Quercetin induces autophagic cell death in HL-60 cells

**Figure 4:**
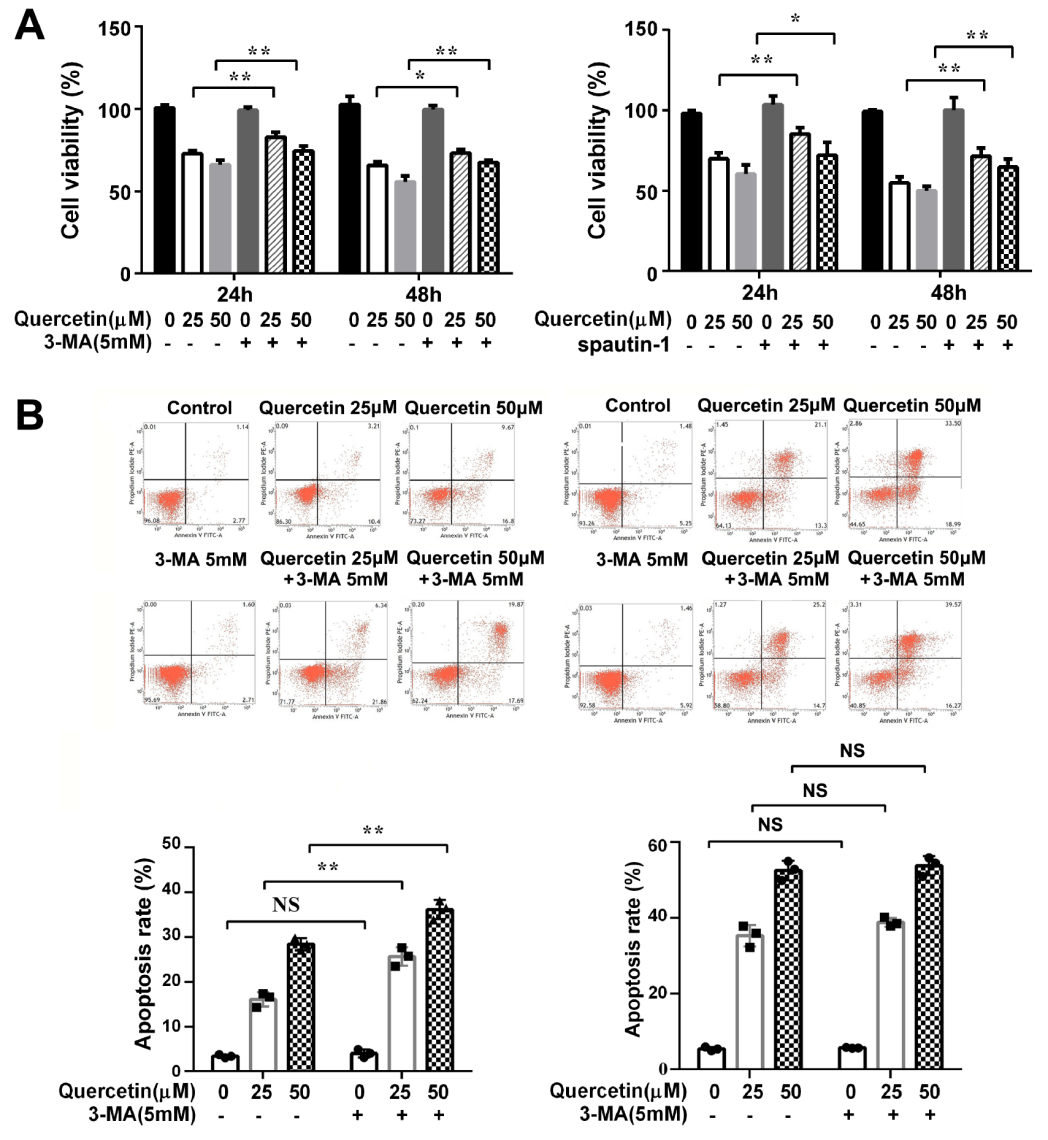
Figure 4 Effect of quercetin on viability and apoptosis of HL-60 cells after autophagy inhibition (A ) The effect of quercetin on cell viability after autophagy inhibition. Cell viability was analyzed by CCK-8 assay. Data are presented as the mean±SD of 4 independent experiments. * P<0.05, ** P<0.01. (B ) The effect of quercetin on cell apoptosis after autophagy inhibition. HL-60 cells were pre-incubated with 3-MA for 4 h, followed by treatment with quercetin for 24 h (Left) and 48 h (Right). The cells apoptosis rates were measured by flow cytometry. The early apoptosis was defined as PI (+) AnnexinV-FITC (–); late apoptosis was defined as PI (+) AnnexinV-FITC (+). ** P<0.01 vs quercetin 25 μM group, * P<0.05 vs quercetin 50 μM group, n=4 for each group. NS, no significance.

The viability and apoptosis rates of HL-60 cells were detected after autophagy was inhibited. The results of CCK-8 assay showed that the cell viability in the quercetin+3-MA or quercetin+spautin-1 group was higher than that in quercetin alone group at both 24 h and 48 h ( Figure 4A), implying that quercetin could induce autophagic cell death in HL-60 cells. After the autophagy was inhibited with 3-MA, flow cytometry was used to analyze cell apoptosis rate. The cell apoptosis in the quercetin+3-MA group was slightly increased at 24 h. However, the difference of cell apoptosis between quercetin+3-MA group and quercetin alone group was not significant at 48 h ( Figure 4B).

We propose that quercetin may play an anti-leukemia role through inducing cell apoptosis and autophagy simultaneously. When the autophagy pathway is blocked, the apoptotic pathway is slightly enhanced. However, this compensatory mechanism will fade as the incubation time is prolonged.

### TAK-1, AMPK and mTOR are potential therapeutic targets for AML

**Figure 5:**
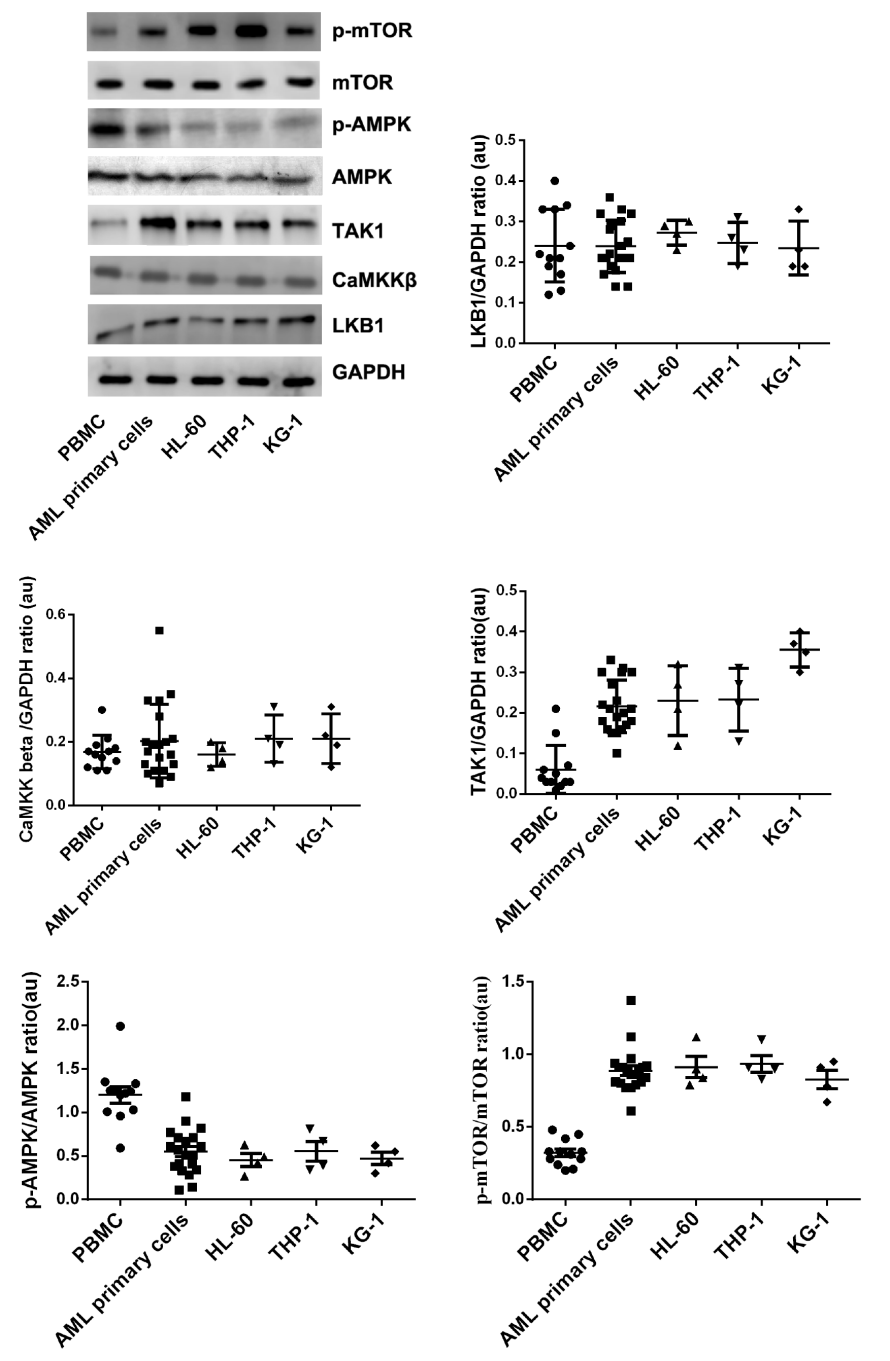
Figure 5 LKB-1, CaMKKβ, TAK-1, AMPK, and mTOR protein expressions in PBMCs, AML primary cells, HL-60 cells, THP-1 cells and KG-1 cells Protein expressions were detected by western blot analysis. n=13 for PBMCs group, n=20 for AML primary cells group, n=4 for HL-60 cells, THP-1 cells and KG-1 cells groups.

The protein expressions of LKB-1, CaMKKβ, TAK-1, AMPK and mTOR in PBMCs, AML primary cells, HL-60 cells, THP-1cells and KG-1 cells were detected by western blot anlysis. The results of variance analysis showed that the expressions of p-AMPK in AML primary cells and AML cell lines were lower than that in normal human PBMCs, and the expressions of p-mTOR in the primary AML cells and AML cell lines were higher than that in normal human PBMCs. These results indicated that inducing the phosphorylation of AMPK or inhibiting the mTOR activity in leukemia cells might be a potential method for acute leukemia treatment. It was also found that LKB-1, CaMKKβ and TAK1 were all expressed in HL-60 cells, primary AML cells and normal human PBMCs. The differences of LKB-1 and CaMKKβ protein expressions among PBMCs, primary AML cells and AML cell lines were not significant. The TAK1 expressions in primary AML cells and AML cell lines were higher than that in PBMCs ( Figure 5). These data showed that regulating the expression of TAK-1 and the phosphorylation of AMPK or/and mTOR might be potential approaches for the treatment of AML.

### Quercetin induces autophagy and apoptosis in anAMPK/mTOR-dependent manner

**Figure 6:**
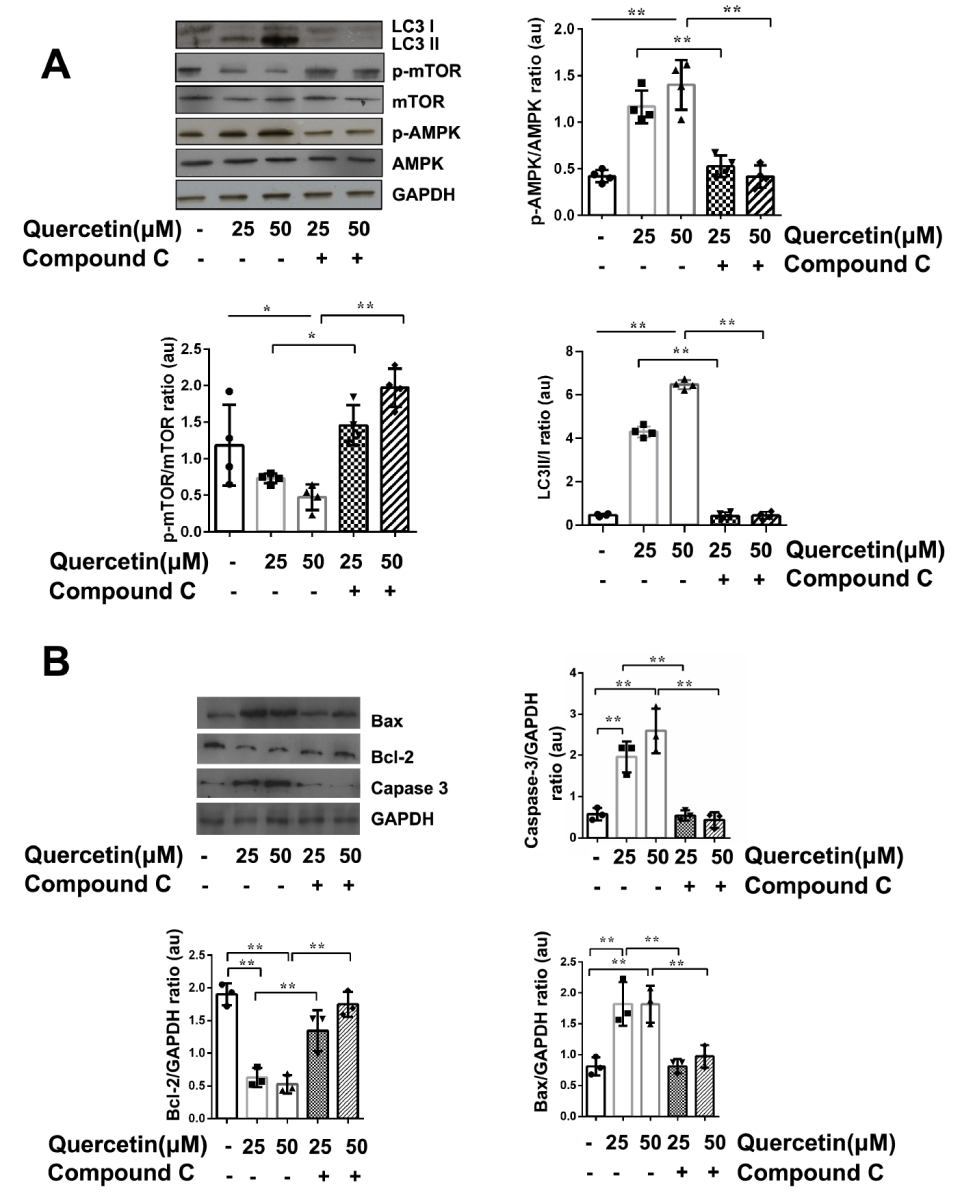
Figure 6 Quercetin induces autophagy and apoptosis in HL-60 cells through the AMPK/mTOR signal pathway HL-60 cells were incubated with quercetin with or without compound C for 48 h. Proteins were isolated and subjected to western blot analysis using indicated antibodies and quantified using Image-pro plus 6. (A) One of four independent experiments is shown. p-AMPK/AMPK, p-mTOR/mTOR and LC3 II/I ratios were expressed in arbitrary units. n=4 for each group. (B) Caspase-3, Bcl-2 and BAX proteins were detected by western blot analysis and expressed in arbitrary units. n=3 for each group. Data are presented as the mean±SD. * P<0.05, ** P<0.01.

We have proven that when the concentration of compound C (an AMPK specific small molecule inhibitor) was less than 50 μM, it would not affect the viability of HL-60 cells. Compound C could specifically inhibit the activity of AMPK at a concentration of over 10 μM [8]. Compound C (25 μM) together with quercetin (25, 50 μM) were chosen to treat HL-60 cells in this study. The expressions of AMPK, p-AMPK, mTOR andp-mTOR were detected by western blot analysis. It was found that quercetin could activate AMPK activity in a concentration-dependent manner. Quercetin increased the phosphorylation level of AMPK in HL-60 cells and decreased the phosphorylation of mTOR. However, while the quercetin-induced phosphorylation of AMPK was inhibited by compound C, the p-mTOR expression was elevated ( Figure 6A). The ratio of LC3 II/I was increased in quercetin-treated cells, suggesting that the level of autophagy was increased, with an obvious increasing trend as the concentration of quercetin was increased. When compound C was used to inhibit quercetin–induced phosphorylation of AMPK, the ratio of LC3 II/I was decreased consequently ( Figure 6A).

The expressions of caspase-3 and Bax were increased in HL-60 cells after treatment with quercetin. In contrast, Bcl-2 expression was decreased. These results suggested that quercetin could induce apoptosis of HL-60 cells. However, when the phosphorylation of AMPK was inhibited by compound C, the effect of quercetin on inducing apoptosis was eliminated ( Figure 6B).

These results suggested that quercetin could inhibit the p-mTOR expression through activating AMPK. When the AMPK’s phosphorylation was inhibited by compound C, the effect of quercetin on mTOR was weakened. Meanwhile, the autophagy and apoptosis levels induced by quercetin were decreased. We propose that quercetin may induce autophagy and apoptosis through activating the phosphorylation AMPK and reducing the consequent phosphorylation of mTOR protein.

### CaMKKβ manipulates the quercetin-induced AMPK phosphorylation and autophagy in HL-60 cells

**Figure 7:**
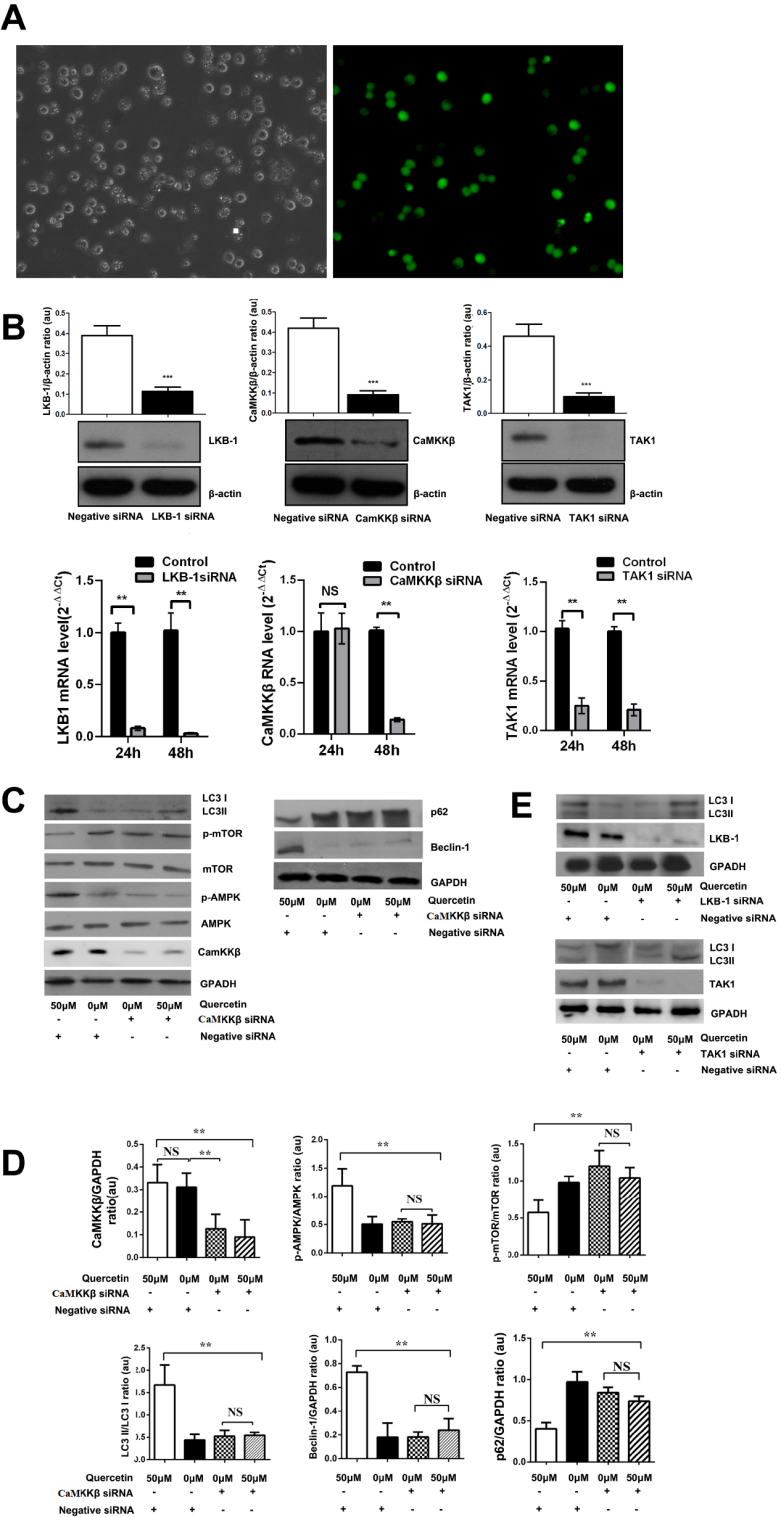
Figure 7 CaMKKβ siRNA transfection and the protein expressions in HL-60 cells (A) Gene silence effect of siRNA. HL-60 cells were transfected with negative control siRNA. Cells were analyzed by fluorescence microscopy (magnification, 200×). (B) LKB-1, CaMKKβ, TAK-1 proteins in HL-60 cells transfected with siRNA were detected by western blot analysis and quantified using Image-pro plus 6. One of three independent experiments is shown. Data are presented as the mean±SD of 3 independent experiments. qRT-PCR analysis of LKB-1, CaMKKβ and TAK-1 mRNA expression in HL-60 cells transfected with siRNA. n=3 for each group. ** P<0.01 vs control group (negative control siRNA group). (C) Protein expressions in CaMKKβ-silenced HL-60 cells. Proteins were isolated and subjected to western blot analysis using indicated antibodies. One of three independent experiments is shown. (D) The proteins were quantified by Image-pro plus 6 and expressed in arbitrary units. Data are presented as the mean±SD of 3 independent experiments. (E) The expressions of LC3 II/I in LKB-1 or TAK-1 silenced HL-60 cells were detected by western blot analysis. * P<0.05, ** P<0.01. NS, no significance.

The upstream kinases of AMPK include LKB1, CaMKKβ and TAK1 [ 19– 21] . In this study, we found that that LKB-1, CaMKKβ and TAK1 were all expressed in HL-60 cells. Therefore, LKB-1 siRNA, CaMKKβ siRNA and TAK1 siRNA were transfected into HL-60 cells respectively. IF staining observed by microscopy visualized that HL-60 cells were effectively transfected with FAM-labeled siRNAs 24 h after Nucleofection ( Figure 7A). Realtime PCR and western blot analysis were used to detect their mRNA and protein expressions. After the cells were transfected for 48 h, the relative protein expressions of LKB-1, CaMKKβ and TAK1 were decreased by 73.54%±3.27%, 64.62%±10.86%, and 65.16%±13.99%, compared to the negative control; and their mRNA levels were decreased by 97.73%±0.25%, 86.04%±1.99% and 79.29%±2.93%, compared to the negative control ( Figure 7B).

HL-60 cells were transfected with CaMKKβ siRNA for 24 h, and then incubated with quercetin for another 48 h. After that, proteins were isolated and subjected to western blot analysis using indicated antibodies. The results showed that 50 μM of quercetin did not affect the CaMKKβ protein expression. CaMKKβ siRNA could inhibit the protein expression of CaMKKβ effectively. There was an interactive effect of quercetin and CaMKKβ siRNA on the protein expressions of p-AMPK and p-mTOR in HL-60 cells. When the *CaMKKβ* gene was silenced in HL-60 cells, the quercetin-activated AMPK phosphorylation was eliminated, and its ability to inactivate mTOR was reduced. Quercetin could induce the increase of LC3 II/I ratio in HL-60 cells. Meanwhile, it could elevate the expression of beclin-1 and reduce the expression of p62 ( Figure 7C,D).

These data supported the idea that quercetin could induce autophagy in HL-60 cells. However, CaMKKβ siRNA eliminated the effect of quercetin on LC3 II/I ratio, beclin-1 protein expression, and p62 protein expression. However, neither LKB-1 nor TAK1 siRNA affected the quercetin-elevated ratio of LC3 II/I ( Figure 7E). We speculate that quercetin may activate autophagy in HL-60 cells through the CaMKKβ/AMPK/mTOR signaling pathway. CaMKKβ might be an upstream signal molecule of quercetin-activated AMPK/mTOR.

## Discussion

In this study, we investigated the anti-leukemic mechanism of quercetin. We found that quercetin could induce cell apoptosis and autophagic cell death, and finally suppress the viability of leukemia cells. Mechanistically, AMPK/mTOR is a vital intracellular signal pathway for quercetin-induced apoptosis and autophagy in leukemic HL-60 cells, while CaMKKβ is an important upstream signaling molecule for AMPK/mTOR pathway.

AML is one of the most common malignancies of the hematopoietic progenitor cell in adults. The cure and survival rate of AML are still not satisfactory [ 23, 24] . To improve the cure rate of AML and prolong the survival of patients, scientists have been searching for new treatments [25]. Quercetin, belonging to flavonoids, is an almost non-toxic medicine. The structure of quercetin is stable and quercetin is easy to be separated and purified. It has gained recognition over the years due to its antioxidant and anti-cancer effects [ 26, 27] .

Tumorigenesis is closely regulated by apoptosis and autophagy [ 28, 29] . The function of autophagy in cell survival is still ambiguous. Some studies found that autophagy played a protective role in the cell survival [ 23– 25] , while others concluded that autophagy was another type of programmed cell death (PCD) [ 26– 28] . Our preliminary study proved that quercetin had anti-leukemia effect by inducing AML cells apoptosis [8]. However, could quercetin affect autophagy in leukemia cells? And if yes, what kind of role would autophagy play in the procedure of leukemia cell death? These questions are unclear so far. Using electron microscopy, immunofluorescence microscopy and flow cytometry assays, we demonstrated that autophagy could be induced by quercetin in the leukemia cells. We also found that the quercetin-induced apoptosis was increased slightly when autophagy was blocked. Combined with our previous research [8], we concluded that quercetin might play the anti-leukemia role through inducing apoptosis and autophagy simultaneously; as quercetin-induced autophagy and apoptosis acted complementarily on the death of HL-60 cells. When the autophagy pathway was blocked by 3-MA, the quercetin-induced apoptosis showed a compensatory increase in the early stage. However, this compensatory mechanism was incomplete and subject to time constraint.

The intracellular signaling pathway by which quercetin induces apoptosis and autophagy in leukemia cells is still unclear. Both apoptosis and autophagy are regulated by intracellular energy metabolism, and some studies have shown that there may be a common signaling pathway for apoptosis and autophagy [ 29, 30] . AMPK is the cellular energy receptor that regulates cell energy metabolism [31]. Tumor is often accompanied by energy metabolism disorder and AMPK activation inhibition [32]. Therefore, AMPK is considered as a potential target for the treatment of tumors [33]. mTOR is a kind of silk/threonine protein kinase. It is an important downstream signal molecule of AMPK and participates in the progress of tumors [34]. Over-activation of mTOR can accelerate the cell cycle of tumor cells and promote cell migration and apoptosis-resistance [ 35– 37] . Green *et al*. [38] found that a specific inhibitor of mTOR induces a multisite dephosphorylation of 4E-BP1, which markedly inhibits the initiation step of mRNA translation, resulting in a strong anti-leukemic activity against primary AML cells while sparing normal hematopoiesis *ex vivo* and significantly reducing the growth of AML cells in nude mice. Studies showed that the regulation of apoptosis and autophagy is intimately connected, and the same regulators can sometimes control both of these two processes [ 39, 40] . mTOR may have multi-effect on the regulation of cell death, including apoptosis and autophagy [41]. The mechanisms linking autophagy and apoptosis are not fully interpreted [42]. Recent studies have revealed that some apoptotic proteins ( *e.g.*, Bax and Bim) modulate autophagy [ 43, 44] . Moreover, autophagic proteins regulate intrinsic apoptosis through calpain- and caspase-mediated cleavage of autophagy-related proteins, which switches the cellular program from autophagy to apoptosis [ 45, 46] . On one hand, autophagy degrades damaged mitochondria and caspases [ 47, 48] , on the other hand, it provides a membrane-based intracellular platform for caspase processing in the regulation of apoptosis [49]. Here, we found that the expressions of phosphorylated AMPK in AML primary cells and HL-60 cells were lower than those in normal human PBMCs, and the expressions of p-mTOR in the primary AML cells and HL-60 cells were higher than those in PBMCs. These results indicated that inducing the phosphorylation of AMPK or inhibiting the mTOR activity in leukemia cells might be a potential method for acute leukemia treatment. Moreover, we observed that p-AMPK was activated and p-mTOR expression was decreased in HL-60 cells under the impact of quercetin. However, AMPK inhibitor compound C could antagonize the above effects of quercetin. Quercetin-induced autophagy and apoptosis levels were decreased when the phosphorylation of AMPK was blocked by compound C. Since the mechanism of quercetin′s anti-leukemia effect is related to AMPK/mTOR signaling pathway, we speculate that quercetin may activate AMPK and inhibit the phosphorylation of mTOR, thus inducing autophagy and apoptosis in the HL-60 cells. In our study, when autophagy was inhibited by 3-MA or spautin-1, cell apoptosis rate was increased. Indeed, our results indicated that quercetin decreased the expression of p62, an autophagy related protein which binds with and activates caspase 8 to induce cell apoptosis [50]. However, further study is needed to clarify the crosslink between quercetin-induced autophagy and apoptosis and the role of AMPK/mTOR in these programmed cell death processes.

As a type of allosteric molecule, the complete activation of AMPK also requires the phosphorylation of the threonine (threonine 172) in its catalytic center by the upstream kinases [51]. The upstream kinases of AMPK include LKB1, CaMKKβ and TAK-1 [ 19– 21] . When cellular energy crisis occurs, LKB1 phosphorylates AMPK, and then inhibits mTOR activity by binding directly to mTOR′s key subunit receptor or through phosphorylating TSC2 [ 52, 53] . In addition to LKB-1, CaMKKβ and TAK1 are also important upstream signal molecules for AMPK. When the concentration of Ca ^2+^ in the cell increases, AMPK can be activated by CaMKKβ [54]. Recent studies have shown that it plays an important role in many kinds of malignant tumors, such as breast cancer, neck tumor and prostate cancer [ 55– 57] . TAK1 could induce autophagic death and be activated by factors such as TLRs, IL-1, TNF and TGF-β [ 58– 60] . How TAK1 causes autophagy is not completely clear, and studies show that it may be realized through the AMPK/mTOR signaling pathway [61]. In this study, we found that LKB-1, CaMKKβ and TAK1 were all expressed in HL-60 cells, primary AML cells and PBMCs. The expression levels of LKB-1 and CaMKKβ protein among PBMCs, primary AML cells and HL-60 cells were not significantly different. The TAK1 expressions in primary AML cells and HL-60 cells were higher than those in PBMCs. When CaMKKβ gene was silenced by siRNA, the function of quercetin on regulating AMPK and mTOR phosphorylation was weakened, and the quercetin-induced autophagy was decreased. This phenomenon was not observed when *LKB-1* or *TAK1* was silenced. We suspected that CaMKKβ might be the upstream molecular of AMPK/mTOR in response to quercetin in HL-60 cells. However, quercetin did not affect the protein expression of CaMKKβ. It has been proven that the protein structure of CaMKKβ could change to an active conformation to bind and form a complex with AMPK under external stimuli. Subsequently, the intracellular concentration of Ca ^2+^ is elevated and finally AMPK is activated [ 62– 64] . We speculate that quercetin may activate AMPK through CaMKKβ without affecting the protein expression of CaMKKβ. Further studies are needed to clarify how quercetin affects the function of CaMKKβ.

Based on our experimental results, we conclude that quercetin has anti-leukemia function, and may induce apoptosis and autophagy of HL-60 cells through modulating the CaMKKβ/AMPK/ mTOR signaling pathway. Our data shed light on the development of potential new strategies for leukemia treatment.
